# Orphans in the Market: The History of Orphan Drug Policy

**DOI:** 10.1093/shm/hkx098

**Published:** 2017-11-27

**Authors:** Koichi Mikami

**Keywords:** orphan drugs, rare diseases, pharmaceutical market, drug regulation, United States

## Abstract

This paper examines the history of orphan drug policy, from the emergence of ‘orphans’ in the American pharmaceutical market in the 1960s, through the debates and agitations that resulted in the passage of the US Orphan Drug Act of 1983, to attempts in the 1990s to prevent abuse of that Act and restore its original intentions. Although an increased number of drugs for rare diseases have since been developed and marketed, the extremely high price of some such drugs is considered a major public health issue internationally. The present paper traces the origins of this issue to the market-based approach to resolving the problem of orphan drugs embodied in the 1983 Act. The paper also makes visible an alternative trajectory that existed for a while in the United Kingdom but was eventually abandoned in order to help the biotechnology industry grow in the context of an increasingly integrated European drug market.

## Introduction

This paper examines the history of orphan drug policy, especially in relation to the US Orphan Drug Act of 1983. The Act is often considered ‘one of the most successful pieces of health related legislation passed in the United States’.^1^ Its author, Representative Henry A. Waxman (D–CA) proudly describes it as ‘an example of government at its finest, demonstrating how Congress applies itself to solve overlooked, but deeply important, problems that affect millions of Americans’.^2^ However, a common criticism of the law is that it has resulted in extremely high prices for some orphan drugs.^3^ By closely examining the longer history of orphan-drug legislation—not just the passage of the 1983 law but the three decades from the early 1960s, when the word ‘orphan’ was first used to describe a class of drugs, through the early 1990s, when orphan drugs were shown to be profitable, this paper presents a new perspective on both what has been achieved and also not achieved by adopting the market-oriented approach embodied in the Act.

The impact of the Act is most commonly measured by the number of new treatments for rare diseases developed after its passage. Whereas only 10 drugs for such diseases had been approved by the US Food and Drug Administration (FDA) in the decade before 1983, by 2010, the agency had approved more than 350.^4^ Orphan products, which barely existed before the 1980s, now account for about a third of all newly-approved drugs and biologics.^5^ The legislation has also served as a model for law or policy internationally, inspiring similar enactments in the 1990s in Singapore, Japan, Australia and Europe. Some scholars view the legislation as creating a new ‘field’ or ‘agora’, in which multiple stakeholders with a shared interest in developing orphan drugs interact and cooperate—a development they view as a major achievement.^6^

The empowerment of patients living with rare diseases is also commonly cited by health care professionals working in the field of rare diseases and others as a major achievement of the Orphan Drug Act, which patient advocacy was crucial in passing.^7^ The impact of the Act on the lives of patients and their families has been expressed, both by individuals who took part in its passage and those who studied it retrospectively, by the idea of ‘hope’. According to Waxman, orphan drugs were once a story of hopelessness.^8^ However, over the last 20 years or so, patient organisations have actively and influentially interacted with physicians and researchers, in their search for curative and ameliorative interventions.^9^

Although individuals who played key roles in the development and passage of the Act as well as scholars writing on the topic have emphasised its benefits—the ways it shifted, in the words of sociologist Carlos Novas, the ‘conduct of firms, industry, regulatory agencies in directions considered socially and economically desirable’—health economists and health policy analysts have increasingly voiced concerns about the prohibitive cost of some orphan drugs.^10^ Although in the 1960s, drugs for rare diseases were ‘orphaned’ due to their lack of profitability, financial and other incentives to their development included in the Act and its modifications resulted in their becoming profitable by the beginning of the 1990s, with some treatments now prohibitively expensive. While Waxman once said that with the Orphan Drug Act ‘we have reordered the economics of pharmaceuticals to make the market work’, the persistent challenge of making drugs for rare diseases available for patients indicates that the orphan drug market has yet to be made to work as well for them as is commonly assumed.^11^ The aim of this paper, which is based on research in primary and secondary sources, including the records of Congressional hearings, and on a series of interviews with key individuals, is primarily to elucidate the historical contingency of the market-oriented approach to solving the problems of orphan drugs, and demonstrate the ways it was shaped by concerns other than addressing rare diseases and saving lives of those affected.

Historical accounts of the Orphan Drug Act, whether related by those who played key roles in the political negotiations leading to its enactment or by others, have tended to present its market-based approach not as a choice but a necessity, reflecting the shared interest of various stakeholders. However, taking into account the entire span of orphan drug law and policy, this paper shows that adoption of a market-based approach to drugs for rare disorders was not a foregone conclusion, and it instead argues that the existence of alternative pathways is obscured when historical accounts focus too narrowly on the discussions that occurred at the beginning of the 1980s.

## Orphaned in Drug Regulation Reform

The idea of some drugs being ‘orphaned’ emerged in the United States when pharmaceutical regulation in that country was undergoing major change after the passage of the 1962 Kefauver–Harris Amendments to the Food, Drug and Cosmetic Act. The bill was originally introduced by Senator Estes Kefauver (D–TN) to increase government control over the pharmaceutical industry and to reduce the price of prescription drugs.^12^ Kefauver’s original bill did not find sufficient support in Senate, but as a consequence of the thalidomide tragedy, a significantly revised version of the bill, emphasising the need to ensure the safety and efficacy of drugs, passed on both floors of Congress in October 1962. Although most of the provisions of the original bill that aimed to increase price competition in the pharmaceutical market had been removed, the Amendments demanded a rigorous but costly approach to clinical trials, authorising the FDA as the responsible government agency to oversee the process of drug development—the ‘gatekeeper’ of the US pharmaceutical market.^13^

Weighing the risk of approving unsafe drugs against that of causing delay in approving life-saving drugs, the FDA decided to emphasise pre-market evaluation in its drug regulation system.^14^ Previously, any drugs for investigational use could be distributed freely to qualified investigators as long as they were labelled as such. Following the Amendments, the FDA issued new regulations stipulating that all drug sponsors would have to submit an investigational new drug (IND) notice before starting clinical trials.^15^ As a transitional measure, the FDA requested that sponsors provide a list of all drugs already undergoing trials, then either submit an IND notice for each of those drugs or withdraw it, informing the FDA of the reasons for doing so.^16^ As a result, a quarter of the drugs initially listed were withdrawn. When the Commission on Drug Safety—a body of experts drawn from industry and academia on pharmaceuticals, set up in 1962 by the Pharmaceutical Manufacturers Association (PMA)—held a conference in Chicago in June 1963, Grant E. Liddle, representative of the American Society for Clinical Investigation, speculated that in most cases the decision to withdraw was due to their ‘low commercial priority’.^17^ While he suggested the regulations might need to be revised, no action followed.

It was also at this conference that Harry C. Shirkey, then chairman of the Committee on Drug Dosage of the American Academy of Pediatrics, first used the word ‘orphan’ in relation to drugs. He spoke of ‘pharmaceutical orphans’ in expressing his concern about an increasing number of drugs that were approved for use in adults, but not in infants and children, leaving children who might benefit from those drugs with fewer therapeutic options.^18^ Shirkey later reasoned that this was due to the small sales potential of such drugs, relative to the cost of obtaining separate FDA approval for paediatric use.^19^ Five years later, George P. Provost, an editor of the *American Journal of Hospital Pharmacy*, invoked the idea of ‘orphans’ to describe substances that were held in hospital pharmacies but not approved for clinical use, despite having been used in preparing medicines before the 1962 Amendments.^20^ Like Shirkey, Provost suspected that the reason the producers did not seek approval was insufficient profitability. Shirkey and Provost both observed that in the past pharmaceutical companies had supplied some drugs at a financial loss as a service to the public—so-called ‘public service drugs’ or ‘service drugs’. Assuming that it was the increased cost of obtaining marketing approval that jeopardised this practice, they argued that doctors and pharmacists should do more to help companies secure approval by collecting relevant information about unprofitable drugs.

The idea of ‘orphan’ was thus first invoked by medical professionals to point out some noticeable impacts of the Kefauver–Harris Amendments, in particular the abandonment of unprofitable drugs by pharmaceutical companies. However, neither the word nor the concern with unprofitable drugs assumed wider currency at that time. Rather, for much of the 1970s, debates about the consequences of drug regulation reform revolved around the concept of ‘drug lag’. Introduced by clinical pharmacologists William M. Wardell and Louis Lasagna, the drug lag debate focused concerns that effective new drugs were reaching the market more slowly in the US market than elsewhere.^21^ Unprofitable drugs were occasionally considered in this context as extreme instances of drug lag, which could only be addressed through ‘the social responsibility of the industry’.^22^ The FDA also paid passing attention to what it called ‘drugs of limited commercial value’ in 1975. Chaired by Marion J. Finkel, then associate director for new drug evaluation at the FDA Bureau of Drugs, the committee contemplated possible incentives to encourage pharmaceutical companies to produce such drugs, but concluded that the reasons they were neglected were too diverse to permit meaningful recommendations.^23^ In effect, they were ‘orphaned’, not just by their manufacturers, but also by those considering drug regulation reform in the United States for much of the 1970s.

## In the Hands of the Nation at Large

A similar problem emerged in the United Kingdom in the mid-1970s. There, however, the problem was addressed very differently from the approach in the United States. As in the USA, the British drug regulation system had undergone considerable change after the thalidomide tragedy.^24^ Initially, a voluntary licensing system was introduced, and the Committee on the Safety of Drugs (CSD) was established in 1963 to the evaluate the safety of drugs, both before and after their marketing. Subsequently, the Medicines Act of 1968 made it mandatory for drug manufacturers to obtain a Clinical Trials Certificate before conducting studies on human subjects and to secure a Product License before marketing any new drug. The Act also stipulated that efficacy as well as safety should be evaluated, while the Medicines Commission replaced the CSD as the government’s expert advisory and drug evaluation body in 1971. Throughout these regulatory changes, the British government maintained a cooperative relationship with domestic drug manufacturers.^25^ Indeed, the British approach received favourable mention in the US ‘drug lag’ debates, which highlighted the greater emphasis in Britain on post-marketing surveillance, as well as the more flexible approach to evaluating efficacy, which primarily relied on evidence submitted by companies while giving clinical practitioners discretion to decide what would be best for their patients.^26^

Nevertheless, at a symposium held in London in 1974, W. H. Lyle of local pharmaceutical manufacturer Dista Products drew attention to the experience of J. M. Walshe at the Department of Investigative Medicine, University of Cambridge.^27^ Walshe had found that triethylene tetramine dihydrochloride (trien) was useful in treating patients with a rare inherited disorder called Wilson’s disease.^28^ He was unable to find a manufacturer willing to make it for him, however, so had to prepare it in his own laboratory. Because the 1968 Medicines Act included an exemption clause that permitted manufacturers to prepare an approved medicine at the request of a medical practitioner if the intention was to administer it only to that practitioner’s patients, Lyle argued that the primary reason for companies’ unwillingness to undertake the production of drugs like trien was the fear of litigation and adverse publicity in the event of mishap, rather than their unprofitability.^29^ He concluded that if progress in treating rare diseases was to be made, ‘vocal and sustained encouragement inside and outside the commercial sphere would be essential’, stating that ‘the remedy [for Walshe’s problem] lies in the hands of the nation at large’.^30^ Supporting Lyle’s argument, an editorial in *The Lancet* suggested that the problem was ‘a matter to which the Government and the Medicines Commission should give prompt consideration.^’^^31^

Walshe himself raised the issue again a year later, arguing in the *British Medical Journal* that because of tight regulation on new drugs after the thalidomide disaster and the resultant high cost of obtaining government approval, any new drugs for rare diseases would likely have to face the same fate as trien.^32^ Emphasising also the legal risk clinicians face in prescribing unapproved drugs to patients, he called for state intervention. His plea was picked up by the *New Scientist* magazine, which described his case as illustrative of ‘an intriguing and important defect’ in Britain’s drug production system.^33^ The magazine was subsequently able to report that a solution had been found for Walshe’s case.^34^ In 1978, the UK Department of Health and Social Services (DHSS) obtained a Clinical Trials Certificate for trien, and the DHSS and the Laboratory of the Government Chemist began collaborating with a pharmaceutical manufacturer K&K-Greeff Chemicals to devise an effective production method.^35^ The company started supplying the chemical to Walshe and others on the Clinical Trials Certificate in the same year. In this instance at least, the problem of drugs for rare diseases was resolved through cooperation between the British government and a local pharmaceutical manufacturer.

## Increased Visibility of Patients

Meanwhile, in the United States, members of Congress were becoming aware of pharmaceutical companies’ reluctance to develop drugs of limited commercial value, thanks in part to the effort of affected patients.^36^ An early such instance was the Congressional Commission for the Control of Huntington’s Disease and Its Consequences, created by the Public Health Service Act of 1975 and chaired by Marjorie Guthrie, founder president of a patient support group called the Committee to Combat Huntington’s Disease.^37^ The Commission’s concern that pharmaceutical companies were not interested in diseases affecting relatively small numbers of people, including, but not limited to, Huntington’s disease, was reported to Congress in 1977.^38^ Consequently, when Senator Edward M. Kennedy (D–MA) proposed to set up a National Center for Clinical Pharmacology in his Drug Regulation Reform bill in 1978, he included research and development of ‘drugs of limited commercial value’ among its responsibilities.^39^

Kennedy’s effort spurred the FDA to make a second attempt to study the problem of drugs of limited commercial value, and in 1978 the agency convened an Interagency Task Force, again chaired by Finkel.^40^ Unlike the previous committee, the Task Force argued that it was ‘less important to establish definitive facts and figures than to undertake appropriate action leading to a solution’.^41^ In contrast to the interventionist approach taken in Britain, however, the Task Force considered that the role of the government should ‘be largely catalytic, managerial and supportive, with limited monetary or credit aid’.^42^ The report acknowledged the past supply of service drugs by the pharmaceutical industry, suggesting that it was evidence of the industry’s willingness and capacity to develop such drugs.^43^ Convinced that the increased cost of obtaining FDA approval was the main factor deterring the industry from producing service drugs, the Task Force recommended a programme of financial support for clinical trials, which recipients would have to repay once their drugs reached the market. A new FDA advisory board should also be set up ‘to encourage voluntary industry action as a matter of public interest and … accord appropriate recognition to firms which participate on the basis of humanitarian concern’.^44^ The Task Force was particularly firm on the point that the essential conditions of safety and efficacy for drug approval—developed after the 1962 Kefauver–Harris Amendments—should not be compromised.

In contrast to the British government’s response to Walshe’s trien problem, the FDA’s approach was thus to provide financial and organisational support to pharmaceutical companies to offset the costs of securing marketing approval, and to encourage their voluntary commitment to produce more service drugs. Critics doubted whether this would be sufficient, however. Lasagna, for instance, observed that the FDA had never done anything to cut the costs of drug production, and argued that ‘orphanization’ of potentially useful but unprofitable drugs would likely persist, because their development was ‘blocked through lack of interest on the part of the people and institutions whose commitment is necessary’—not only pharmaceutical companies but also the FDA.^45^

In the event, Kennedy’s bill failed in 1978 and again in 1979. But further interventions from patients and practitioners helped to keep the matter before Congress. Melvin H. Van Woert, a researcher at Mount Sinai Hospital, had developed an effective treatment for myoclonus, a rare neurologic symptom; the treatment included a naturally occurring substance called L-5-hydroxytryptophan (L-5HTP).^46^ Since L-5HTP was not manufactured commercially, Van Woert had to prepare it in his laboratory. He had approached the FDA, the US National Institutes of Health (NIH) and the PMA for help, but none were able to offer a solution. Van Woert and one of his myoclonus patients, Sharon Dobkin, therefore contacted their local Representative Elizabeth Holtzman (D–NY) and convinced her that legislative action was needed. In early 1980, Holtzman introduced a bill outlining measures ‘to assist in the development of drugs for diseases and conditions of low incidence.’^47^ Based on the recommendations of the Task Force, her bill proposed to provide administrative and economic assistance to pharmaceutical companies for the research and development of such drugs.^48^ Meanwhile, Representative Waxman was alerted to a similar issue by the mother of a patient with Tourette syndrome, whose medication had been available in Canada but was not approved in the United States.^49^ A keen advocate of health care reform and recently-appointed chair of the House Subcommittee on Health and the Environment, Waxman arranged for Holtzman’s bill to be heard by the committee in June 1980.

Despite Waxman’s support, Holtzman’s bill did not pass the House of Representatives. However, on the day after the hearing, *The Los Angeles Times* published a small article about it. This caught the eye of Maurice Klugman, who was then suffering from a rare form of cancer, and he and his brother, actor Jack Klugman, produced an episode in the television drama series *Quincy M.D.* based on the story of the family that had asked Waxman for help. The episode was effective in increasing the visibility of patients and families and building public support for legislative effort to address the problem of drugs of limited commercial value, and is remembered by many as the moment when ‘the ball began to roll’.^50^ Although Holtzman lost her Congressional seat soon after, Representative Ted Weiss (D–NY) resubmitted her bill, which received another hearing in 1981. Later that year, Waxman submitted his own bill, which would eventually become the Orphan Drug Act of 1983.

## Orphan Drugs as Cases of Market Failure

The Orphan Drug Act did not simply enact Holtzman’s original proposals, however. Rather, the Congressional deliberations that took place over the various bills led to a profound shift in how the orphan drug problem was conceived. Holtzman’s bill, like the FDA Task Force from which it drew inspiration, saw the problem as one of ensuring that existing drugs were made available to patients. At the first hearing on Holtzman’s bill, Finkel for the FDA accordingly recommended that government support should ‘be provided only for drugs for which some evidence of probable safety and effectiveness already existed’—a criterion that applied to both the myoclonus and Tourette syndrome cases.^51^ Dobkin echoed this view. Speaking from her own experience with L-5HTP, she argued that ‘the worst thing that can happen to a person is to hold a treatment in his hand, see the miracles it can bring, and then have it pulled away.^’^^52^ However, Abbey S. Meyers, who was then vice president of the Tourette Syndrome Association, and had been invited to speak by Waxman, expressed a rather different view.^53^ She stated in her testimony:

Millions of Americans who suffer from rare diseases live without hope. We believe that there are not enough dollars among patients who suffer from sickle cell anemia, Cooley’s anemia, Huntington’s disease, cystic fibrosis, Wilson’s disease, Tay Sachs disease, dystonia, and many, many more, to make manufacture of a therapeutic drug profitable.^54^

Meyers’ statement was based on her communication with other patient groups, whom she had contacted to see if they had faced similar problems to Tourette patients.^55^ Many confirmed that they did—but some went further. Meyers and Dobkin were concerned that beneficial treatments had been taken away from them—‘the worst thing’ that Dobkin described. But some patients suffered from conditions for which no such effective treatment had ever even been developed—and these patients feared that the pharmaceutical industry’s lack of interest in rare diseases meant they never would be. In effect, these patients indicated that the scope of the orphan drug problem needed to be expanded, to include not just the problem of securing access to existing but unapproved drugs, to the larger question of how to promote the development of new drugs for rare conditions.

Waxman was sympathetic to this future-oriented view of the problem, and in opening the second hearing on Holtzman’s bill, he spoke of ‘victims of orphan diseases’ in reference to patients of Tourette syndrome, Huntington’s disease, amyotrophic lateral sclerosis and spina bifida, whose medical needs were ignored by drug manufactures because of the low number of patients affected.^56^ Subsequently, when Waxman brought his own bill forward in place of Holtzman’s, he included measures that were intended not just to ease the way to approval of existing drugs for rare conditions—for instance proposing that a single well-controlled clinical trial, backed up by post-marketing surveillance, should suffice for FDA approval—but also recommending substantial incentives for companies that brought such drugs, both pre-existing and novel, to market.^57^ This included offering tax credits in proportion to the cost of clinical testing of designated orphan drugs, and granting seven-year exclusive marketing rights if the drug were not patentable. By such means, Waxman aimed to cut the cost of developing drugs for rare diseases and provide incentives for their development. His commitment to developing new drugs for neglected conditions was underscored when he invited Marjorie Guthrie, who in 1977 had first brought this very issue to the attention of Congress through the Commission she chaired, to address the hearing on his bill in March 1982.^58^

## Entangled with the Drug Regulation Reform Debate

Waxman’s strategy was in effect to represent the orphan drug problem as one of market failure, attributable to the peculiarly small market for such drugs, and to be addressed by introducing market incentives aimed specifically at such products.^59^ However, his efforts to demarcate the market of orphan drugs from the rest of the US pharmaceutical market initially met with considerable opposition from the pharmaceutical industry.

Through the 1970s, the pharmaceutical industry had repeatedly challenged strict FDA regulations by pointing to the problem of ‘drug lag’, while occasionally citing the problem of drugs for rare conditions as a special case of this larger regulatory problem.^60^ After the *Quincy M.D.* episode increased the public profile of the orphan drug problem, however, pharmaceutical companies began to realise that this might provide an opportunity to draw wider attention to their own concerns. Contrary to Waxman’s understanding of the issue, therefore, their strategy turned on representing orphan drugs, not as a circumscribed problem of market failure, but as epitomising a wider problem of excessive FDA regulation on new drugs.^61^

No one from the industry had attended to testify at the first hearing on Holtzman’s bill. Following the *Quincy M.D.* broadcast, however, Lewis A. Engman, then president of the PMA, along with a few others from the industry, decided to testify at the second hearing in March 1981. Engman explained that the industry had in the past addressed issues of rare diseases by its supply of service drugs, and proposed that the PMA should continue to work to that end by establishing a Commission on Drugs for Rare Diseases, which would act as an information clearinghouse. However, he insisted that what was really needed was not new legislation but improvement in and speeding up of the drug approval process—not just for orphan drugs but for all drugs.^62^ David E. Collins, then president of McNeil Pharmaceutical, supported Engman’s argument, stating explicitly that ‘the orphan drug problem is a significant manifestation of a broader problem relating to drug discovery, development and approval^’^.^63^ Several members of Congress also gave them their support: for example, Representative James H. Scheuer (D–NY) used the phrase ‘regulatory overkill’ to make the same point.^64^

The industry pursued the same line at the 1982 hearing on Waxman’s bill. Peter Barton Hutt, former chief counsel of the FDA but now representing the PMA, again opposed any legislative action aiming specifically at orphan drugs. He first asserted the industry’s commitment to solving the problem by noting its creation of the Commission on Drugs for Rare Diseases earlier that year. He then stated:

In our view there is no statutory inhibition to the development of orphan drugs. Rather the problem seems to be in how the law is implemented during the investigational new drug and new drug application review process. … It is an unnecessarily rigid implementation of the statute that creates problems not only for new orphan drugs, but for all new drugs.^65^

The pharmaceutical industry’s priority was thus to challenge the conventions of FDA regulation in general, while resisting any measures aimed specifically at orphan drugs. Their views were shared by some within the administration itself. Thus where Waxman’s bill proposed to expedite approval of orphan drugs by shifting the regulatory onus from pre-market testing to post-marketing surveillance, Edward Brandt Jr., then associate secretary of health at the DHHS, argued that this measure was unnecessary because the FDA already had discretion to give patients faster access to drugs—whether orphan or non-orphan—as INDs on a compassionate basis.^66^ Meanwhile, the FDA continued to defend its existing regulatory standards, while supporting the idea that special measures should be introduced to tackle the particular problem of orphan drugs. At the second hearing on Holtzman’s bill, for instance, J. Richard Crout, then director of the FDA Bureau of Drugs, reiterated the view that the unmet needs of rare-disease patients represented a failure of the drug market rather than of drug regulation, and ended by reasserting the agency’s policy of applying the same standards of safety and efficacy to both orphan and non-orphan drugs.^67^

Entangled as they now were in the wider political debate on drug regulation reform and the adequacy of the FDA’s approach to regulating the pharmaceutical market, Waxman’s legislative attempt might not have succeeded had he not received support from two other sources. The first of these was the emerging alliance of rare-disease patients and medical researchers. At the hearing on Waxman’s bill, Jess G. Thoene of the University of Michigan, who was at that time running a clinical trial of a chemical component cysteamine in patients with a rare inborn error of metabolism called cystinosis, was impressed by Guthrie’s testimony and invoked it in his own.^68^ After the hearing, Guthrie said to him ‘we are in this together, we cannot let this momentum slide, this is a major issue, let’s work together’.^69^ One of Thoene’s Michigan colleagues—George J. Brewer, who was researching another rare condition called Wilson’s disease—went on to organise a conference in Ann Arbor in September 1982, where plans to establish a coalition for rare diseases took shape.^70^ That coalition—later to be formalised as the National Organization for Rare Disorders (NORD)—was established initially as an *ad hoc* organisation primarily to monitor the progress of Waxman’s orphan drug bill, and help to publicise the issues it addressed.^71^

The second, more unexpected source of support came from generic drug manufacturers. The hearing on Waxman’s bill was happening at the same time as the growing generic drug industry was engaged in its own negotiations over the stringency of FDA regulations.^72^ Founded at the beginning of the 1980s, the new Generic Pharmaceutical Industry Association (GPIA) was trying to secure an ‘abbreviated new drug application’, requiring only proof of bioequivalence rather than full-scale clinical trials for generic versions of drugs, which had previously received FDA approval and were now out of patent.^73^ The FDA had already made this pathway available for generic versions of drugs approved before 1962, and negotiations now focused on more recently approved medicines. The GPIA was working with several legislators, including Waxman, on this issue.^74^ Soon after the hearing on Waxman’s orphan drug bill, GPIA president William F. Haddad realised that one of the reasons why some drugs became orphaned was because they were not patent protected, so pharmaceutical companies were reluctant to invest in them. But off-patent drugs were precisely the territory that the emerging generic sector was trying to claim for itself, while the opportunity to address the orphan drug problem promised favourable publicity for the sector. The GPIA promptly set up the Institute for Orphan Drugs to encourage generic firms to seize this opportunity.^75^

The GPIA’s swift action threatened to outflank the PMA, which was itself fighting for legislation to extend drug patents and so minimise competition from generic manufacturers. Faced with a potential public relations coup, the PMA capitulated and gave its support to Waxman’s bill.^76^ By doing so, the pharmaceutical companies also in effect conceded Waxman’s construal of orphan drugs as cases of market failure, deserving distinctive arrangements from the rest of the drug market. As a result, they were no longer able to claim that orphan drugs were just extreme instances of a wider ‘drug lag’ problem. Meanwhile, having secured support from both consumers and potential suppliers of orphan drugs, Waxman’s bill passed the House of Representatives with minor amendments, and the Orphan Drug Act was finally signed into law by US President Ronald Reagan in January 1983.^77^

## The Machinery of Orphan Drugs

The 1983 Orphan Drug Act retained the provisions for seven-year exclusive marketing rights and tax credits—though reduced now to equal to 50 per cent of the cost of clinical trials—from Waxman’s original bill, while the clause to shift toward the post-marketing surveillance was removed because this was not favoured by neither the FDA nor the pharmaceutical industry.^78^ The Act also authorised the DHHS secretary to offer grants through the FDA for clinical trials of orphan drugs in fiscal years 1983 to 1985.^79^ Despite these measures, however, most pharmaceutical companies remained reluctant to invest in orphan drugs, and while members of the GPIA were more supportive, generic firms did not necessarily have the right expertise for the development of new drugs.^80^ Furthermore, the Reagan administration failed to appropriate the budget for the FDA grants in both 1983 and 1984, and the agency could make only limited funds available for clinical trials of orphan drugs.^81^ Noting these shortcomings, Waxman observed in 1985 that while the passage of the Act had fostered hope, the objective of addressing the orphan drug problem would only be achieved if ‘the tools’ in the Act were adjusted in light of the continuing impediments.^82^ Efforts were made in the mid-1980s to make just such adjustments.

The most significant adjustment made was to define ‘rare diseases’ in a way that clarified the boundary of the orphan drug market. The original Act had specified that in order to qualify for orphan drug designation, the manufacturer needed to set out the ‘facts and circumstances’ that rendered that drug unprofitable.^83^ However, the FDA found it impossible to determine if a drug was eligible for orphan drug designation because the price of the product and the cost of its development—hence the prospect of making a profit or lack thereof—were not included in the information its sponsor conventionally submitted to the agency.^84^ Finkel, in her new capacity as the first director of the FDA Office of Orphan Products Development (OOPD), had been aware of this issue and argued at the 1982 conference in Ann Arbor that it would be necessary to define the upper limits of profitability.^85^ She suggested that a way to resolve this issue might be to specify ‘the number of potential users of a drug’; she proposed ‘an incidence of 0.05%’—that is, a prevalence of 100,000 patients in the United States—as a reasonable line to draw.^86^ The figure was put to representatives of the industry and consumers at a meeting which Meyers attended as chair of NORD’s Government and Industry Liaison Committee. During a break, Meyers took Finkel to the ladies’ room—they were the only female attendees—and recommended that the figure be raised to 200,000 since a threshold of 100,000 would preclude at least three diseases that she knew of—Tourette syndrome, multiple sclerosis and narcolepsy.^87^ The figure of 200,000 was duly adopted as the legal definition of a rare disease in the 1984 Amendments to the Orphan Drug Act.^88^

Further amendments to the Act were made in 1985.^89^ These included expanding eligibility for seven-year market exclusivity to cover patentable as well as non-patentable drugs. At the House Oversight Hearing on the Act held prior to the amendments, the FDA had reported that a number of products had been submitted for orphan drug designation with patents which were due to expire before or soon after obtaining of FDA approval.^90^ Such products were not currently covered by the Act. In order to incentivise development of such products by companies and to prevent the situation where the government had to take on the responsibility for their development, the condition that eligible substances be unpatented was struck out.^91^ The amendments also established the National Commission on Orphan Diseases (NCOD) with the mandate of assessing activities relating to rare diseases undertaken by government agencies—including the NIH and the FDA—as well as those at private organisations.^92^ The NCOD was directed by a former OOPD officer Stephen C. Groft and chaired by the Michigan researcher Jess G. Thoene. Its members also included Van Woert and Meyers, as well as Marlene E. Haffner, who had assumed directorship at the OOPD after Finkel left the FDA.^93^

Despite these adjustments, problems persisted with the working of the Act. Many of the drugs taken forward immediately after passage of the Act were ones for which prior knowledge of their safety and efficacy existed. But Waxman and others were worried that sufficient incentives were not yet in place to stimulate research into new drugs for rare diseases.^94^ In the words of Van Woert, ‘the machinery is now in place to develop known orphan drugs’ but ‘other approaches to stimulate and facilitate preclinical and early clinical research’ were needed for its real success.^95^ These worries were borne out by an NCOD report of 1989 which revealed that of the $1.3 billion that the US government spent on rare disease research in 1987, more than half was spent on approximately 200 rare forms of cancer—mostly studied at the National Cancer Institute—with about $640 million left for the other 4,800 rare diseases.^96^ The report also revealed that many academic investigators believed that getting funding for research on rare diseases was harder to secure than for similar studies on more common diseases.^97^ This was discouraging for those concerned about orphan drugs. Without sufficient government support, the market-based approach to promoting the development of new orphan drugs was not living up to earlier hopes.^98^

## No Orphan in the United Kingdom

Across the Atlantic, the UK government continued to adopt a different approach to drugs for rare diseases. In 1985, the Cambridge researcher J. M. Walshe and I. Herbert Scheinberg of Albert Einstein College of Medicine in New York organised a colloquium on orphan drugs in London. R. D. Mann of the DHSS told the meeting that British regulations posed ‘no regulatory impediment to considering the benefit–risk ratio of an orphan drug in a realistic fashion’.^99^ Under the UK Medicines Act of 1968, the licensing authority had flexibility in assessing a drug’s safety and efficacy, and could take into consideration both the severity of the conditions it was used for and the number of potential users. The Act also permitted doctors to treat patients under their care with unapproved drugs on a ‘named patient’ basis. Directive 75/318 of the European Economic Community (EEC), introduced in 1975, likewise allowed doctors to prescribe products for rare indications even though they might not satisfy the normal data requirements for marketing approval. The British and European regulations thus entrusted medical professionals to evaluate the risks and benefits of drugs for their patients, and to act as gatekeepers for new drugs. Furthermore, where no manufacturer was willing to produce a drug, the Secretary of State in the United Kingdom could hold the relevant Product License in the interest of patients—the approach taken in Walshe’s case in the late 1970s. Indeed, Rosalinde Hurley, who chaired the Medicines Commission between 1982 and 1993 and also served on the management board of the European Medicines Evaluation Agency, went so far as to tell the colloquium: ‘I certainly regard [the term “orphan”] as *most* regrettable’ and ‘would not welcome it into the language of the United Kingdom, certainly not into any statute of this realm’.^100^ Under UK regulations, she implied, there was simply no need for a law comparable to the US Orphan Drug Act.

By the mid-1990s, however, European provision was moving closer to the American model. In 1991, the Research Trust for Metabolic Diseases in Children, a patient organisation for rare diseases in Britain, organised its 10th anniversary conference on the topic of orphan drugs. At that meeting, Robert Hangartner of the UK Department of Health observed that while EEC Directive 75/318 addressed the issue of drugs that could not be tested in standard clinical trials owing to small patient numbers, it made no provision for drugs that were not commercially viable.^101^ European discussion of possible legislation for orphan drugs accordingly came to focus increasingly on that latter point, as moves to integrate the European pharmaceutical market and harmonise drug regulations across Europe gained momentum.^102^ In particular, European policy makers and drug companies in Europe came to favour legislation on the lines of the US Orphan Drug Act when they saw how it assisted the growth of the American biotechnology industry in the early 1990s.^103^

## Birth of Billion-dollar Orphans

By the late 1980s, concerns were being expressed that the market incentives provided under the 1983 Orphan Drug Act and its later amendments were not just encouraging the manufacture of otherwise unprofitable drugs, but were open to ‘abuse’, with some companies making ‘exorbitant profits’ from designated orphan drugs.^104^ Consequently, Congress decided to investigate these concerns, and to evaluate the sustainability of the market-based approach to the orphan drug problem embodied in the Act.

In February 1990, a hearing was held before the House Subcommittee on Health and the Environment, at which three drugs were examined. Two of these were products of the new biotechnology industry: recombinant human growth hormone (r-HGH), marketed independently by Genentech and Eli Lilly, and approved for treatment of children with growth failure; and Amgen’s recombinant erythropoietin (r-EPO), used to treat anaemia associated with chronic renal failure.^105^ The third, aerosol pentamidine, was a conventional drug produced by Lyphomed to treat a type of pneumonia common to AIDS patients. Not only were these drugs expensive; there also existed companies interested in producing competitive products but prevented from doing so by the market-exclusivity clause of the Orphan Drug Act.

At the hearing, Meyers argued that r-HGH was a clear case of abuse, since it would have been developed even without the incentives in the Act.^106^ Genentech and Eli Lilly both started developing the drug in the late 1970s, and only applied for orphan drug designation after the 1985 Amendments extended eligibility to include patentable products.^107^ Since the drug was not a case of market failure, Meyers argued, it should not be eligible for the incentives available under the Act. John McLaughlin, general counsel of Genentech, counter-argued that the company had endeavoured to fill an unmet need by replacing an expensive pituitary drug with the cheaper, though still expensive, recombinant option, and by setting up a free access programme for uninsured patients.^108^ Several members of the Subcommittee also disagreed with Meyers; for example, Representative Scheuer argued that he did not think profiting under the provisions of the Act qualified as a case of abuse.^109^

Aerosol pentamidine and r-EPO presented slightly different issues. Aerosol pentamidine was designated an orphan drug at a time when few AIDS patients had been identified. With the growth of the epidemic and hence the potential market, other companies wished to develop cheaper versions of the same drug, but the market exclusivity provisions of the Act prevented such competition. In the case of r-EPO, orphan drug status was granted on grounds of the rarity of the particular condition specified by its sponsor, despite knowledge that the drug could also be useful for a number of other indications—an approach that Gabriel Schmergel, then president of a competitor company The Genetic Institute, criticised as ‘salami slicing’.^110^

Consumers were alarmed at the high price of these drugs, but warned that any solution had to be devised in such a way that companies remained interested in developing orphan drugs. At the hearing, several approaches were considered. One—the idea of allowing ‘shared exclusivity’ to replace market monopoly with oligopolistic competition—was rejected because it would render the market-exclusivity provision of the Orphan Drug Act practically meaningless. Another was to control the price by setting an upper limit on the sales that a company could accrue from a drug before its orphan drug designation was rescinded, but this too was rejected: the logic of the market-based approach was to offer incentives in the form of potential profits, while it would be politically problematic to intervene in pricing decisions. Ironically labelling the drugs discussed at the hearing ‘billion-dollar orphans’, one reporter concluded that there was little prospect of a ‘miracle cure’ that would make both suppliers and consumers of orphan drugs happy.^111^

Nonetheless, later in 1990, Congress passed further amendments to the Orphan Drug Act which permitted shared exclusivity where a company could prove that their product was developed simultaneously to a designated orphan drug, and which mandated withdrawal of orphan drug status if the targeted population exceeded 200,000. The amendments were, however, pocket-vetoed by then US President George H. W. Bush, a staunch proponent of the free market.^112^ Thoene, chair of the NCOD and also president of NORD at that time, voiced his concern that ‘the future of the Orphan Drug Act is unclear’ and that any ‘cure’ must involve distinguishing ‘true’ orphan drugs from ‘psuedo-orphans’.^113^

A further attempt to cure the Act was made in 1991, with an amending bill that set an upper limit of $200 million on the sales of orphan drugs. The bill was heard before the Senate Subcommittees on Antitrust, Monopolies and Business Rights and on Labor and Human Resources. This time, the hearings focused on a drug called alglucerase, approved in April 1991 and sold by Genzyme for treatment of Gaucher’s disease; its estimated sales for the first 10 months were $120 million. At the hearing, Meyers again argued that the Act was not intended to help companies developing blockbuster products but to address cases of market failure.^114^ Some rare diseases organisations disagreed, however. Robert Dresing, then president of the Cystic Fibrosis Foundation, argued that rare-disease patients needed ‘the power and force of the high technology and the sophistication that only the pharmaceutical and biotech industries could supply’, insisting that the Act was serving its purpose.^115^ Strong dissent was also expressed by Senator Orrin G. Hatch (R–UT), for whom the Orphan Drug Act was ‘a success story’.^116^ In his view, the amendments bill was ‘a perfect example of a misguided effort to fix a law that isn’t broken’.^117^ Hatch also emphasised the importance of the Act to the still nascent biotechnology industry.^118^ His argument was endorsed by Henri A. Termeer, then chief executive officer of Genzyme, who stated that for young biotechnology firms, like his, ‘the orphan area is extremely important and extremely attractive’.^119^ The bill died in Congress without a vote, and no cure was found for the Orphan Drug Act. Meanwhile, biotechnology companies continued to benefit. In 1994, Genzyme introduced a recombinant version of alglucerase, selling it at the same price as the earlier product while significantly reducing production costs. The case made clear that even ‘true orphans’ can be profitable, provided high enough prices could be extracted from consumers.^120^

Thus, while it was evident that some companies made large profits from orphan drugs, Congressional deliberations in the 1990s left the market-based approach intact. As a result, ‘billion-dollar orphans’ multiplied and became increasingly widely accepted. Orphan drugs were no longer ‘drugs of limited commercial value’ but attractive business opportunities, while patients with rare diseases were no longer ‘victims’ of orphan diseases but ‘consumers’ in the potentially lucrative orphan drug market.

## Discussion

The Orphan Drug Act of 1983 is commonly recognised as an important piece of legislation that for the first time addressed issues of rare diseases and conditions, which had long been neglected in the drug market. The idea of ‘hope’—used by Waxman and others to justify their legislative efforts and sometimes echoed in scholarly commentaries—emphasises the positive impact of the Act on the lives of those affected.^121^ From being ‘without hope’, those affected by rare diseases now have reason to hope that drugs will be developed—a change that is also evident in their transformation from ‘victims’ of orphan diseases to ‘consumers’ of orphan products.^122^ However, as Christine Hayes of the Huntington’s Disease Society of America said in 1992, ‘hope is no good if a drug is developed and our folks cannot afford it’.^123^ The ongoing discussion about the extremely high price of some orphan drugs suggests that for some people living with rare diseases, the hope they have been given may turn out to be a false one.

This paper has endeavoured to unravel this conundrum through social historical analysis. It has characterised the orphan drug policy developed in the United States as market-based, in that it sought to solve the problem of orphan drug development by creating market incentives to encourage pharmaceutical companies and biotechnology firms to invest in producing new drugs, while avoiding interference in the pharmaceutical market more generally. This approach stands in contrast to the one initially taken in the United Kingdom, which also sought to mobilise industry, but through direct government involvement.

The market-based approach came to dominate in the United States in part because the FDA adhered to its policy on the ‘risk versus risk’ dilemma in drug regulation, insisting that pre-marketing evaluation should apply equally to orphan and non-orphan drugs.^124^ While Waxman’s bill initially included a clause to place greater emphasis on post-marketing surveillance as part of its special arrangements for orphan drugs, it was removed before the Act was signed into law. In part, too, the decision to extend the seven-year market exclusivity provision to patentable drugs reflected the conviction that the FDA—being the gatekeeper of the US drug market—should make minimal interventions in drug development. The market-based approach to the orphan drug problem thus reflected a peculiarly American understanding of the regulatory role of the FDA at that time, just as the UK approach reflected the more cooperative regulatory culture that existed in Britain.

To an extent, other countries came to regard the US approach to the orphan drug problem as a successful model, adopting aspects of a market-based approach into their own policies in the 1990s, in part because they saw it as effective in assisting the growth of the nascent biotechnology industry. The interaction between biotechnological innovation and orphan drug provision deserves further research. By the late 1980s, biotechnology startups were benefiting from the Orphan Drug Act, not least because securing orphan drug designation for their still-in-development products served as a milestone in their progress—signalling the promise of future profits—for their investors.^125^ This, too, had benefits for patients with rare diseases. The biotechnology industry has done rather more to address some of the unmet medical needs of patients with rare diseases than has the pharmaceutical and generic drug industries; while efforts to emulate the growth of the American biotechnology industry helped to drive the adoption of orphan drug legislation—including epidemiological definitions of ‘rare diseases’—in other countries.^126^

However, it is important to realise that the market-based approach has generated a rather different political economy of drugs for rare diseases than was initially envisaged. This point can be illustrated by comparing the functions of ‘service drugs’ with the more recent turn to ‘free access programmes’ for those who cannot afford highly-priced medicines. Both can be seen as voluntary and humanitarian acts by which companies provide drugs to needy patients. However, while the former utilises resources that a pharmaceutical company gains from its sales of non-orphan products to fund the provision of unprofitable medicines, the latter diverts part of the profits from an expensive orphan drug to make the same drug available to patients who cannot afford it. Manufacturers of billion-dollar orphans, including Genzyme, have also gone on to develop products for other rare diseases and conditions—but this too was financed by profits made primarily on earlier orphan drugs. So long as the main producers of orphan drugs are relatively small biotechnology firms specialised in rare diseases, therefore, helping rare-diseases patients will continue to depend on allowing those firms to profit sufficiently from the small numbers of patients who desperately need their products.^127^ In effect, the hope that those affected by rare diseases now invest in the development of new drugs depends on the promise of future profits. Meanwhile, the growing involvement of large pharmaceutical companies in orphan drugs suggests an even less anticipated outcome, as the profits from orphan products now come to fund the development of drugs for more common diseases.

In 1986, Waxman stated that ‘The Orphan Drug Act is meant to demonstrate that society puts a higher value on helping victims of rare diseases than does the pharmaceutical market place.’^128^ Now, however, the question has become whether society can afford the prices determined by the orphan drug market. Thus, the problem of orphan drugs—like that of ‘drug disasters’—thus presents a valuable case for examining the lasting impact of drug regulation reform in the late twentieth century, and for evaluating whether the current system of drug development and regulation actually serves to maximise the interests of patients and public health.^129^

## Footnotes

1 Carlos Novas, ‘Orphan Drugs, Patient Activism and Contemporary Healthcare’, *Quaderni*, 2009, 68, 13–23, 18.

2 Henry A. Waxman, *The Waxman Report: How Congress Really Works* (New York: Twelve, 2009), 73.

3 Marlene E. Haffner, Josep Torrent-Farnell and Paul D. Maher, ‘Does Orphan Drug Legislation Really Answer the Needs of Patients?’, *The Lancet*, 2008, 371, 2041–4; see also André Côté and Bernard Keating, ‘What Is Wrong with Orphan Drug Policies?’, *Value in Health*, 2012, 15, 1185–91; Emily A. Largent and Steven D. Pearson, ‘Which Orphans Will Find a Home?: The Rule of Rescue in Resource Allocation for Rare Diseases’, *Hastings Center Report*, 2012, 42, 27–34; Olivier Wellman-Labadie and Youwen Zhou, ‘The US Orphan Drug Act: Rare Disease Research Stimulator or Commercial Opportunity?’, *Health Policy*, 2010, 95, 216–28.

4 Marilyn J. Field and Thomas F. Boat, eds, *Rare Diseases and Orphan Products: Accelerating Research and Development* (Washington, DC: National Academy Press, 2010), 2.

5 Largent and Pearson, ‘Which Orphans’, 28.

6 Helen Crompton, ‘Mode 2 Knowledge Production: Evidence from Orphan Drug Networks’, *Science and Public Policy*, 2007, 34, 199–211; Karim Hamadache, ‘Reintroducing Power and Struggles within Organisational Fields: A Return to Bourdieu’s Framework’, in Ahu Tatli, Mustafa Ozbiligin and Mine Karatas-Ozkan, eds, *Pierre Bourdieu, Organization and Management* (London: Routledge, 2015), 97–118; Karim Hamadache and Julienne Brabet, ‘Rethinking Institutional Entrepreneurship: The Case of the Construction of the Orphan Drug Field in Europe’, *Society and Business Review*, 2014, 9, 139–52.

7 Ségolène Aymé, Anne Kole and Stephen Groft, ‘Empowerment of Patients: Lessons from the Rare Diseases Community’, *The Lancet*, 2008, 371, 2048–51, 2050; see also Caroline Huyard, ‘How Did Uncommon Disorders became “Rare Diseases”?’, *Sociology of Health & Illness*, 2009, 31, 463–77; Novas, ‘Orphan Drugs’.

8 US Congress House Committee on Energy and Commerce, *FDA Issues: Hearings before the Subcommittee on Health and the Environment*, 99^th^ Congress, 1st Session (Washington, DC: US Government Printing Office, 1985), 709.

9 Carlos Novas, ‘The Political Economy of Hope: Patients’ Organizations, Science and Biovalue, *BioSocieties*, 2006, 1, 289–305; Carlos Novas, ‘Patients, Profits and Values: Myozyme as an Exemplar of Biosociality’, in Sahra Gibbon and Carlos Novas, eds, *Biosocialities, Genetics and the Social Sciences: Making Biologies and Identities* (London: Routledge, 2008), 136–56.

10 Novas, ‘Orphan Drugs’, 17; see also Côté and Keating, ‘Orphan Drug Policies’; Largent and Pearson, ‘Which Orphans’.

11 Henry A. Waxman, ‘The History and Development of the Orphan Drug Act’, in I. Herbert Scheinberg and J. M. Walshe, eds, *Orphan Diseases and Orphan Drugs* (Manchester: Manchester University Press, 1986), 135–49, 136.

12 Carolyn H. Asbury, *Orphan Drugs: Medical versus Market Value* (Lexington, MA: Lexington Books, 1985); Daniel Carpenter, *Reputation and Power: Organizational Image and Pharmaceutical Regulation at the FDA* (Princeton, NJ: Princeton University Press, 2010); Dominique A. Tobbell, *Pills, Power, and Policy: The Struggle for Drug Reform in Cold War America and its Consequences* (Berkeley & Los Angeles, CA: University of California Press, 2012).

13 Carpenter, *Reputation and Power*; see also John Abraham, *Science, Politics and the Pharmaceutical Industry: Controversy and Bias in Drug Regulation* (London: UCL Press, 1995); Arthur A. Daemmrich, *Pharmacopolitics: Drug Regulation in the United States and Germany* (Chapel Hill, NC: the University of North Carolina Press, 2004); Lucas Richert, *Conservatism, Consumer Choice, and the Food and Drug Administration during the Reagan Era: A Prescription for Scandal* (Lexington, MA: Lexington Books, 2014); Tobbell, *Pills, Power and Policy*.

14 Arthur Daemmrich, ‘A Tale of Two Experts: Thalidomide and Political Engagement in the United States and West Germany’, *Social History of Medicine*, 2002, 15, 137–58; Arthur Daemmrich and Georg Krücken, ‘Risk Versus Risk: Decision-making Dilemmas of Drug Regulation in the United States and Germany’, *Science as Culture*, 2000, 9, 505–34; see also Frances O. Kelsey, ‘Thalidomide Update: Regulatory Aspects’, *Teratology*, 1988, 38, 221–6.

15 Asbury, *Orphan Drugs*.

16 Frances O. Kelsey. ‘The Investigational Drug Branch: A Review of Objectives and Function’, in Commission on Drug Safety, ed., *Proceedings: Conference of Professional Societies* (Chicago, IL: Commission on Drug Safety, 1963), 104–18.

17 Commission on Drug Safety, ‘Impact of the Investigational New Drug Regulations on the Research Process: A Panel’, in Commission on Drug Safety, ed., *Proceedings: Conference of Professional Societies* (Chicago, IL: Commission on Drug Safety, 1963), 140–56, 144. For the Commission on Drug Safety, see Tobbell, *Pills, Power, and Policy*, 130–3.

18 Commission on Drug Safety, ‘Activities of the Professional and Scientific Societies in Drug Safety: A Panel’, in Commission on Drug Safety, ed., *Proceedings: Conference of Professional Societies* (Chicago, IL: Commission on Drug Safety, 1963), 9–35, 18.

19 Harry Shirkey, ‘Editorial Comment: Therapeutic Orphans’, *The Journal of Pediatrics*, 1968, 72, 119–20.

20 George P. Provost, ‘Editorial: “Homeless” or “Orphan” Drugs’, *American Journal of Hospital Pharmacy*, 1968, 25, 609.

21 William M. Wardell, ‘Introduction of New Therapeutic Drugs in the United States and Great Britain: An International Comparison’, *Clinical Pharmacology and Therapeutics*, 1973, 14, 773–90; William M. Wardell, ‘Therapeutic Implications of the Drug Lag’, *Clinical Pharmacology and Therapeutics,* 1974, 15, 73–96; William M. Wardell and Louis Lasagna, *Regulation and Drug Developmen*t (Washington, DC: American Enterprise Institute for Public Policy Research, 1975); see also Arthur Daemmrich, ‘Invisible Monuments and the Costs of Pharmaceutical Regulation: Twenty-Five Years of Drug Lag Debate’, *Pharmacy in History*, 2003, 45, 3–17; Edward Nik-Khah, ‘Neoliberal Pharmaceutical Science and the Chicago School of Economics’, *Social Studies of Science*, 2014, 44, 489–517; Tobbell, *Pills, Power, and Policy*.

22 Henry G. Grabowski, *Drug Regulation and Innovation: Empirical Evidence and Policy Options* (Washington, DC: American Enterprise Institute for Public Policy Research, 1976), 53.

23 Interagency Task Force on Significant Drugs of Limited Commercial Value, *Significant Drugs of Limited Commercial Value: Report of Interagency Task Force to the Secretary of Health, Education, and Welfare*, 29 June 1979, Appendix 2 ‘Committee on Drugs of Limited Commercial Values Interim Report—May 14, 1975’, A9–A48. Having joined the FDA in 1963, Finkel had considerable influence on the practice of drug review at the agency in the 1970s, also initiating efforts to prioritise innovative drugs over others and quickly approve drugs already approved in other countries. See Carpenter, *Reputation and Powe*r, 485–533.

24 Abraham, *Pharmaceutical Industry*; Stephen J. Ceccoli, ‘Divergent Paths to Drug Regulation in the United States and the United Kingdom’, *Journal of Policy History*, 2002, 14, 135–69; R. D. Mann, ‘From Mithridatium to Modern Medicine: The Management of Drug Safety’, *Journal of the Royal Society of Medicine*, 1988, 81, 725–8.

25 Abraham, *Pharmaceutical Industry*; John Abraham and Courtney Davis, ‘Testing Times: The Emergence of the Practolol Disaster and its Challenge to British Drug Regulation in the Modern Period’, *Social History of Medicine*, 2006, 19, 127–47.

26 Ceccoli, ‘Divergent Paths’; Wardell, ‘An International Comparison’; Wardell, ‘Therapeutic Implications’; Wardell and Lasagna, *Drug Developmen*t; see also John Abraham and Courtney Davis, ‘Deficits, Expectations and Paradigms in British and American Drug Safety Assessments: Prising Open the Black Box of Regulatory Science’, *Science, Technology & Human Values*, 2007, 32, 399–431.

27 W. H. Lyle, ‘Drugs for Rare Diseases’, *Postgraduate Medical Journal*, 1974, 50, 107–8; Anonymous, ‘Drugs for Rare Diseases: Whose Responsibility?’, *The Lancet*, 1974, 303, 440.

28 J. M. Walshe, ‘Management of Penicillamine Nephropathy in Wilson’s Disease: A New Chelating Agent’, *The Lancet*, 1969, 294, 1401–2; J. M. Walshe, ‘Copper Chelation in Patients with Wilson’s Disease’, *Quarterly Journal of Medicine*, 1972, XLII, 441–52.

29 Dista Products was one of the drug manufacturers Walshe approached. See J. M. Walshe, ‘Triethylene Tetramine Dihydrochloride: A New Chelating Agent for Copper’, in Fred E. Karch, ed., *Orphan Drugs* (New York: Marcel Dekker, Inc., 1982), 57–71, 66.

30 Lyle, ‘Drugs for Rare Diseases’, 108.

31 Anonymous, ‘Rare Diseases’, 440.

32 J. M. Walshe, ‘Drugs for Rare Diseases’, *British Medical Journal*, 1975, 3, 701–2.

33 Donald Gould, ‘Drugs for Rare Diseases’, *New Scientist*, 1975, 67, 693.

34 Donald Gould, ‘… and rare diseases’, *New Scientist*, 1978, 78, 346.

35 Trien was granted a Product License in 1985. Roger Humphreys and Rupert Purchase, ‘The Development of Trientine Dihydrochloride, 1977–1985’, in I. Herbert Scheinberg and J. M. Walshe, eds, *Orphan Diseases and Orphan Drugs* (Manchester: Manchester University Press, 1986), 53–5.

36 See Daemmrich, ‘Invisible Monuments’.

37 For the work of patient organisations for Huntington’s disease in the 1970s, see Alice Wexler, *Mapping Fate: A Memoir of Family, Risk, and Genetic Research* (Berkeley & Los Angeles, CA: University of California Press, 1995).

38 Commission for the Control of Huntington’s Disease and its Consequences, *Report: Commission for the Control of Huntington’s Disease and its Consequences, Volume I—Overview* (Washington, DC: US Department of Health, Education, and Welfare, 1977); US Congress Senate Committee on Appropriations, *Special Oversight Hearing: Hearing before the Subcommittee on Labor, Health, Education, and Welfare and Related Agencies*, 95th Congress, 1st Session (Washington, DC: Government Printing Office, 1977); see also Anonymous, ‘Drug Companies Accused of Lagging in Research to Curb Rare Diseases’, *New York Times*, 18 October 1977, 34.

39 US Congress Senate Committee on Human Resources, *Drug Regulation Reform Act of 1978: Hearings before the Subcommittee on Health and Scientific Research*, 95th Congress, 2nd Session (Washington, DC: Government Printing Office, 1978), 6; see also Tobbell, *Pills, Power, and Policy*, 182.

40 Marion J. Finkel, ‘Special Report: Drugs of Limited Commercial Value’, *The New England Journal of Medicine*, 1980, 302, 643–4; Interagency Task Force, *Report*.

41 Interagency Task Force, *Report*, 13.

42
*Ibid.*, 26.

43 Finkel, ‘Special Report’.

44 Interagency Task Force, *Report*, 3.

45 Louis Lasagna, ‘Who Will Adopt the Orphan Drugs’, *Regulation: AEI Journal on Government and Society*, 1979, 3, 221–4.

46 Melvin H. Van Woert, ‘Profitable and Nonprofitable Drugs’, *The New England Journal of Medicine*, 1978, 296, 903–6.

47 US Congress House Committee on Interstate and Foreign Commerce, *Drug Regulation Reform—Oversight Orphan Drugs: Hearing before the Subcommittee on Health and the Environment*, 96th Congress, 2nd Session (Washington, DC: Government Printing Office, 1980).

48 Because of her emphasis on R&D activities, Holtzman’s bill proposed to establish an office to coordinate such activities at the NIH, instead of the advisory board at the FDA. See Asbury, *Orphan Drugs*, 122–3.

49 Waxman, *Waxman Report*; Waxman, ‘Orphan Drug Act’. For the history of Tourette syndrome, see Howard I. Kushner, *A Cursing Brain?: The Histories of Tourette Syndrome* (Cambridge, MA: Harvard University Press, 1999).

50 Abbey S. Meyers, ‘Orphan Drugs and Orphan Diseases: The Consumer’s Viewpoint’, in George J. Brewer, ed., *Orphan Drugs and Orphan Diseases: Clinical Realities and Public Policy* (New York, NY: Alan R. Liss, 1983), 149–57, 151; see also Asbury, *Orphan Drugs*; Hamadache, ‘Organisational Fields’; Huyard, ‘Uncommon Disorders’; Novas, ‘Orphan Drugs’; Waxman, *Waxman Report*.

51 House Committee, *Drug Regulation Reform*, 96th Congress, 31. For Tourette syndrome, a drug called Pimozide had been released outside the United States, and some patients in the country had had access to it as an IND for a short while too. McNeil Pharmaceutical, the company holding the US marketing rights for the drug, applied for FDA approval to market it for schizophrenia, but the FDA turned down the application requesting completion of a two-year animal study to prove the drug’s safety. At that point, the company decided not to pursue the case any further because there were other drugs for schizophrenia and the potential market seemed limited. Pimozide thus became unavailable in the United States.

52
*Ibid.*, 11.

53 For the history of the Tourette Syndrome Association, see Kushner, *Cursing Brain*, 176–87.

54 House Committee, *Drug Regulation Reform*, 96th Congress, 20.

55 Abbey S. Meyers, interview, 29 January 2015; see also Hamadache, ‘Organizational Fields’.

56 US Congress House Committee on Energy and Commerce, *Health and the Environment Miscellaneous—Part 2: Hearings before the Subcommittee on Health and the Environment*, 97th Congress, 1st Session (Washington, DC: US Government Printing Office, 1981), 2.

57 US Congress House Committee on Energy and Commerce, *Orphan Drug Act: Hearings before the Subcommittee on Health and the Environment*, 97th Congress, 2nd Session (Washington, DC: US Government Printing Office, 1982), 268; See also Asbury, *Orphan Drugs*.

58 House Committee, *Orphan Drug Act*, 97th Congress. After passage of the National Sickle Cell Anemia Control Act and the similar law on thalassemia in 1972, a number of patient groups approached their local Congresspersons to have legislation specifically for their diseases, resulting in a proliferation of bills concerning individual genetic diseases in the early 1970s. In this context, Guthrie deliberately took ‘a generic approach’ to rare genetic diseases in order to avoid her Commission’s findings being perceived as ‘a narrow parochial concern’. Charles McKay, interview, 26 February 2015. McKay was deputy director of the Commission.

59 See Waxman, ‘Orphan Drug Act’, 135–6.

60 E.g. James B. Russo, ‘Profitable and Nonprofitable Drugs’, *The New England Journal of Medicine*, 1978, 299, 156; See also Lasanga, ‘Orphan Drugs’.

61 See Thomas Althus, ‘Orphan Drugs—Debunking a Myth’, *The New England Journal of Medicine*, 1980, 303, 1004–5.

62 House Committee, *Miscellaneous*, 97th Congress, 76.

63
*Ibid.*, 136. For McNeil Pharmaceutical, see note 51.

64
*Ibid.*, 15.

65 House Committee, *Orphan Drug Act*, 97th Congress, 285.

66
*Ibid.*, 319.

67 House Committee, *Miscellaneous*, 97th Congress, 28. For J. Richard Crout, see Carpenter, *Reputation and Power*, 487–92; Richert, *The Reagan Era*, 66–8.

68 House Committee, *Orphan Drug Act*, 97th Congress, 349.

69 Jess G. Thoene, interview, 5 February 2015. Guthrie was an inspirational figure to many individuals who were running patient organisations at that time, including Meyers. Meyers, interview.

70 George J. Brewer, interview, 5 February 2015; see also Asbury, *Orphan Drugs*; George J. Brewer, ed., *Orphan Drugs and Orphan Diseases: Clinical Realities and Public Policy* (New York: Alan R. Liss, 1983); Meyers, ‘Consumer’s viewpoint’.

71 Mary Dunkle, ‘A 30-year Retrospective: National Organization for Rare Disorders, the Orphan Drug Act, and the Role of Rare Disease Patient Advocacy Groups’, *Orphan Drugs: Research and Reviews*, 2014, 4, 19–27; Abbey S. Meyers, ‘Summary Roundtable II: The Orphan Drug Problem from the Perspective of the Consumer’, in George J. Brewer, ed., *Orphan Drugs and Orphan Diseases: Clinical Realities and Public Policy* (New York: Alan R. Liss, 1983), 239–42. Not all ‘victims of orphan diseases’ welcomed the establishment of the coalition; for example, the Muscular Dystrophy Association, which had already been successful in fundraising and increasing public awareness through its telethon programme, did not wish to be considered ‘one of many’ and hence did not initially join the coalition. Meyers, interview; see also Meyers, ‘Consumer’s viewpoint’.

72 Jeremy A. Greene, *Generic: The Unbranding of Modern Medicine* (Baltimore, MD: John Hopkins University Press, 2014).

73 For the idea of bioequivalence, *ibid.*, 121–31; see also Daniel Carpenter and Dominique A. Tobbell, ‘Bioequivalence: The Regulatory Career of a Pharmaceutical Concept’, *Bulletin of the History of Medicine*, 2011, 85, 93–131.

74 Greene, *Generic*, 87.

75 Asbury, *Orphan Drugs*, 160–1; see also William Haddad, ‘Generic Pharmaceutical Industry Association Orphan Drug Institute’, in Melvin H. Van Woert and Eunyong Chung, eds, *Cooperative Approaches to Research and Development of Orphan Drugs* (New York: Alan R. Liss, 1985), 41–5.

76 For the debate on patent extension, see Greene, *Generic*; Richert, *The Reagan Era*, 133–40.

77 Waxman, ‘Orphan Drug Act’.

78 The clause was not favoured by the pharmaceutical industry because it felt that stating that only a single clinical trial would be required for orphan drugs could indicate that two trials were necessary for other drugs. Asbury, *Orphan Drugs*, 161–2.

79 Orphan Drug Act of 1983, *Public Law 97–414, US Statutes at Large 99*, 1983, 2049–66; see also Asbury, *Orphan Drugs*.

80 House Committee, *Miscellaneous*, 98th Congress; Brewer, interview; Meyers, interview; Thoene, interview; see also Asbury, *Orphan Drugs*.

81 House Committee, *Miscellaneous*, 98th Congress. For general impacts of the Reagan administration on the US drug policy, see Richert, *The Reagan Era*.

82 Waxman, ‘Orphan Drug Act’, 136.

83 Orphan Drug Act, *Public Law 97–414*, 2050.

84 Stephen C. Groft, interview, 3 March 2015. Groft worked with Finkel at the FDA before becoming executive director of the National Commission on Orphan Diseases in 1985 and later director of the Office of Rare Diseases Research at the NIH.

85 Marion J. Finkel, ‘Orphan Products: Definition and Activities’, in George J. Brewer, ed., *Orphan Drugs and Orphan Diseases: Clinical Realities and Public Policy* (New York: Alan R. Liss, 1983), 159–71, 163. The FDA established the OOPD in 1982 to respond to the PMA’s establishment of the Commission on Drugs for Rare Diseases and demonstrate its willingness to address the orphan drug problem.

86
*Ibid.*, 163; see also Marion J. Finkel, ‘The Orphan Drug Act and the Federal Government’s Orphan Products Development Program’, *Public Health Reports*, 1984, 99, 313–16.

87 Meyers, interview.

88 Health Promotion and Disease Prevention Amendments of 1984, *Public Law 98–551, US Statutes at Large 98*, 1984, 2815–22.

89 Orphan Drug Amendments of 1985, *Public Law 99–91*, US Statutes at Large 99, 1985.

90 House Committee, *FDA Issues*, 99th Congress, 722.

91 See Waxman, ‘Orphan Drug Act’, 144.

92 Orphan Drug Amendments, *Public Law 99–91*; see also Waxman, *‘*Orphan Drug Act’, 143–4.

93 Finkel joined a company called Barlex Laboratories. See Marion J. Finkel, ‘The US Orphan Drug Development Programme: Incentives and Results’, in I. Herbert Scheinberg and J. M. Walshe, eds, *Orphan Diseases and Orphan Drugs* (Manchester: Manchester University Press, 1986), 159–68.

94 House Committee, *Miscellaneous*, 98th Congress.

95 Melvin H. Van Woert, ‘Introduction’, in Melvin H. Van Woert and Eunyong Chung, eds, *Cooperative Approaches to Research and Development of Orphan Drugs* (New York: Alan R. Liss, 1985), 3–6, 5.

96 National Commission on Orphan Diseases, *Report of the National Commission on Orphan Diseases* (Washington, DC: US Department of Health and Human Services, 1989), 41.

97
*Ibid.*, xiv.

98 Some disease-specific patient groups managed to secure support from the NIH for research on their disease, but their success was the exception rather than the rule. See Ruby Horansky, ‘The Role of Voluntary Disease Organizations in Research & Development of Orphan Drugs’, in Melvin H. Van Woert and Eunyong Chung, eds, *Cooperative Approaches to Research and Development of Orphan Drugs* (New York: Alan R. Liss, 1985), 179–81; for a successful case, Dominique A. Tobbell, ‘Charitable Innovations: The Political Economy of Thalassemia Research and Drug Development in the United States, 1960–2000’, in Viviane Quirke and Judy Slinn, eds, *Perspectives on Twentieth-Century Pharmaceuticals* (Bern: Peter Lang, 2010), 302–37.

99 R. D. Mann, ‘UK Regulatory Requirements for Orphan Drugs’, in I. Herbert Scheinberg and J. M. Walshe, eds, *Orphan Diseases and Orphan Drugs* (Manchester: Manchester University Press, 1986), 146–9, 147.

100 William H. Bachrach *et al.*, ‘Round-table Discussion’, in I. Herbert Scheinberg and J. M. Walshe, eds, *Orphan Diseases and Orphan Drugs* (Manchester: Manchester University Press, 1986), 204–15, 204.

101 Research Trust for Metabolic Diseases in Children, ‘Orphan Diseases and Orphan Drugs: A Report Based on the Panel Discussion, RTMDC Conference’, *RTMDC News*, 1991, 3, 5–8.

102 For integration of the European pharmaceutical market, see John Abraham and Graham Lewis, *Regulating Medicine in Europe: Competition, Expertise and Public Health* (London: Routledge, 2000).

103 Lesley Greene, interview, 20 October 2015. Greene is founder of the Research Trust for Metabolic Diseases in Children and also a founder member of a European coalition organisation for rare diseases called EURORDIS. For European discussion on orphan drug regulation, see Crompton, ‘Mode 2’; Hamadache and Brabet, ‘institutional entrepreneurship’; Huyard, ‘uncommon disorders’.

104 National Commission, *Report*, 57.

105 US Congress House Committee on Energy and Commerce, *Orphan Drug Act: Hearing before the Subcommittee on Health and the Environment*, 101st Congress, 2nd Session (Washington, DC: US Government Printing Office, 1990), 1.

106
*Ibid.*, 31.

107 For development of r-HGH, see Nicolas Rasmusseen, *Gene Jockeys: Life Science and the Rise of Biotech Enterprise* (Baltimore, MD: Johns Hopkins University Press, 2014), 72–100.

108 House Committee, *Orphan Drug Act*, 101st Congress, 113.

109
*Ibid.*, 63.

110
*Ibid.*, 193. For fierce competition in development of r-EPO, see Rasmusseen, *Gene Jockeys*, 131–59.

111 Ann Gibbons, ‘Billion-Dollar Orphans: Prescription for Trouble’, *Science*, 1990, 248, 678–9, 679.

112 Waxman, *Waxman Report*; see also Richert, *The Reagan Era*.

113 Jess G. Thoene, ‘Curing the Orphan Drug Act’, *Science*, 1991, 251, 1158–9.

114 US Congress Senate Committee on the Judiciary, *Anticompetitive Abuse of the Orphan Drug Act: Invitation to High Prices: Hearing before the Subcommittee on Antitrust, Monopolies and Business Rights*, 102nd Congress, 2nd Session (Washington, DC: US Government Printing Office, 1992), 135.

115 US Congress Senate Committee on Labor and Human Resources, *Orphan Drug Amendments of 1991: Hearing before the Committee on Labor and Human Resources*, 102nd Congress, 2nd Session (Washington, DC: US Government Printing Office, 1992), 65. For the Cystic Fibrosis Foundation’s search for cure, see Alan Stockdale, ‘Waiting for the Cure: Mapping the Social Relations of Human Gene Therapy Research’, *Sociology of Health & Illness*, 1999, 21, 579–96.

116 Senate Committee, *Orphan Drug Amendments*, 102nd Congress, 18.

117 Senate Committee, *Anticompetitive Abuse*, 102nd Congress, 5.

118 Senate Committee, *Orphan Drug Amendments*, 102nd Congress, 20.

119 Senate Committee, *Anticompetitive Abuse*, 102nd Congress, 186.

120 US Office of Technology Assessment, *Federal and Private Roles in the Development and Provision of Alglucerase Therapy for Gaucher Disease* (Washington, DC: US Government Printing Office, 1992); Novas, ‘Biosociality’, 147; see also Alan M. Garber, ‘No Price Too High?’, *The New England Journal of Medicine*, 1992, 327, 1676–8.

121 See Novas, ‘Political Economy’; Novas, ‘Biosociality’.

122 House Committee, *Miscellaneous*, 96th Congress, 20.

123 Senate Committee, *Orphan Drug Amendments*, 102nd Congress. Meyers also stated that if the drug were not affordable or inaccessible, for patients it would be the same as if it were ‘never invented’. Senate Committee, *Anticompetitive Abuse*, 102nd Congress, 134.

124 The FDA later became more sympathetic to post-marketing surveillance. See Daemmrich, ‘Invisible Monuments’; Daemmrich, *Pharmacopolitics*; Daemmrich and Krücken, ‘Risk Versus Risk’.

125 For business strategy of biotechnology firms, see Louis Galambos and Jeffrey L. Sturchio, ‘Pharmaceutical Firms and the Transition to Biotechnology: A Study in Strategic Innovation’, *The Business History Review*, 1998, 72, 250–78.

126 Huyard, ‘Uncommon Disorders’; Novas, *‘*Orphan Drugs’.

127 Côté and Keating, ‘Orphan Drug Policies’; Largent and Pearson, ‘Which Orphans’; Wellman-Labadie and Zhou, ‘Orphan Drug Act’.

128 Waxman, ‘Orphan Drug Act’, 136.

129 Abraham, *Pharmaceutical Industry*; Daemmrich, *Pharmacopolitics*; see also John Abraham, ‘Sociology of Pharmaceutical Development and Regulation: A Realist Empirical Research Programme’, *Sociology of Health & Illness*, 2008, 30, 869–85.

## Notes

Dr Koichi Mikami is a visiting research fellow with the Making Genomic Medicine project, led by Professor Steve Sturdy at the University of Edinburgh. He is responsible for its research strand entitled ‘the rise of rare diseases’, and his current research investigates the roles of patients in shaping the rare disease field.

